# Aberrant expression of the candidate tumor suppressor gene DAL-1 due to hypermethylation in gastric cancer

**DOI:** 10.1038/srep21755

**Published:** 2016-02-29

**Authors:** Hao Wang, Man Xu, Xiaobo Cui, Yixin Liu, Yi Zhang, Yu Sui, Dong Wang, Lei Peng, Dexu Wang, Jingcui Yu

**Affiliations:** 1 Scientific Research Centre, the Second Affiliated Hospital of Harbin Medical University, Harbin 150081, China; 2 Laboratory of Medical Genetics, Harbin Medical University, Harbin 150081, China; 3 Department of Hepatopancreatobiliary Surgery, the Second Affiliated Hospital of Harbin Medical University, Harbin 150081, China; 4 Computer teaching and research section, School of Basic Medical Science, Harbin Medical University, China

## Abstract

By allelotyping for loss of heterozygosity (LOH), we previously identified a deletion region that harbors the candidate tumor suppressor gene DAL-1 at 18p11.3 in sporadic gastric cancers (GCs). The expression and function of DAL-1 in GCs remained unclear. Here, we demonstrated that the absence of or notable decreases in the expression of DAL-1 mRNA and protein was highly correlated with CpG hypermethylation of the DAL-1 promoter in primary GC tissues and in GC cell lines. Furthermore, abnormal DAL-1 subcellular localization was also observed in GC cells. Exogenous DAL-1 effectively inhibited cancer cell proliferation, migration, invasion and epithelial to mesenchymal transition (EMT); exogenous DAL-1 also promoted apoptosis in GC AGS cells. When endogenous DAL-1 was knocked down in GC HGC-27 cells, the cells appeared highly aggressive. Taken together, these findings provide solid evidence that aberrant expression of DAL-1 by hypermethylation in the promoter region results in tumor suppressor gene behavior that plays important roles in the malignancy of GCs. Understanding the role of it played in the molecular pathogenesis of GC, DAL-1 might be a potential biomarker for molecular diagnosis and evaluation of the GC.

Gastric cancer (GC) is the fifth most common cancer in the world, nearly 1.0 million new cases were diagnosed in 2012. The identification of the vital molecules related to gastric carcinogenesis is very meaningful. Our previous allelotyping for loss of heterozygosity (LOH) using 14 polymorphic microsatellite markers first described LOH at 18p11.3 in 45 sporadic GCs, suggesting that the 18p11.3 region may be comprised of candidate tumor suppressor genes that are found within the deleted band^1^. The differentially expressed in adenocarcinoma of the lung-1 (DAL-1), also known as erythrocyte membrane protein band 4.1-like 3 (EPB41L3) or 4.1B, is localized to the chromosomal region 18p11.3; this region is affected by LOH in lung, brain, and breast cancers^2^. DAL-1, which belongs to the protein 4.1 superfamily, was first isolated as an expressed fragment of the 4.1 gene by differential display analysis of primary adenocarcinomas of the lung by Tran *et al.* DAL-1 is expressed in various normal tissues; however, its expression is greatly reduced or lost in lung^3^, breast^4^, prostate^5^, and kidney^6^ cancers and in meningiomas^7^. The restoration of DAL–1 expression in non-small cell lung carcinoma (NSCLC) and in breast cancer cells significantly suppressed cell growth *in vitro*^3^,^4^. Although LOH on 18p11.3 was reported in approximately 40% of NSCLC^2^ cases and in 60-70% of meningiomas^7^ and breast cancers^8^, it was determined that LOH in this region was not correlated with loss of DAL-1 expression^7^. Moreover, mutational screening failed to identify inactivating mutations of DAL-1^9^. Several studies have demonstrated the epigenetic inactivation of DAL-1 in some cancers. DAL-1 methylation in renal, lung, and breast cancers and in nasal NK/T-cell lymphoma was found to be associated with the downregulation of DAL-1 expression. Methylation has been shown to be an important mechanism of transcriptional silencing of this gene in these cancers^6^,^10^,^11^,^12^. These findings indicate that DAL-1 is a candidate tumor suppressor gene and may serve as a target for inactivation in carcinogenesis.

Like other 4.1 protein family members, DAL-1 localizes to the plasma membrane near points of cell-cell contact. DAL-1 also suppresses growth of lung cancer cells^3^. DAL-1 directly interacts with tumor suppressor in lung cancer 1 (TSLC1), whose juxtamembrane portion acts as a 4.1-binding motif. Both DAL-1 and TSLC1 are spectrin-actin-binding proteins. The redistribution of both DAL-1 and TSLC1 to newly generated membrane ruffling areas indicates that these proteins are also involved in the cell motility that accompanies actin rearrangement^13^. Migration and invasive behaviors are important characteristics of cancer cells. Such behaviors indicate malignancy, supporting the view that both DAL-1 and TSLC1 can be targeted for the development of anti-cancer agents^14^. To the best of our knowledge, the DAL-1 gene has not yet been characterized in GC.

Our previous study described 18p11.3 (DAL-1 locus) LOH in sporadic GCs^1^, suggesting that DAL-1 may be a candidate tumor suppressor gene in GC. To further investigate the significance of DAL-1 in gastric carcinogenesis, we analyzed the expression and methylation status of DAL-1 in eight GC cell lines and 38 surgically resected primary GCs. We found significant DAL-1 alterations in these GCs and promoter methylation-mediated downregulation of DAL-1, which appeared to be involved in GC pathogenesis. We also demonstrated through functional analyses that the aberrant expression of DAL-1 is associated with malignancy in GC cells.

## Results

### Downregulation of DAL-1 expression in GCs

**Figure 1:**
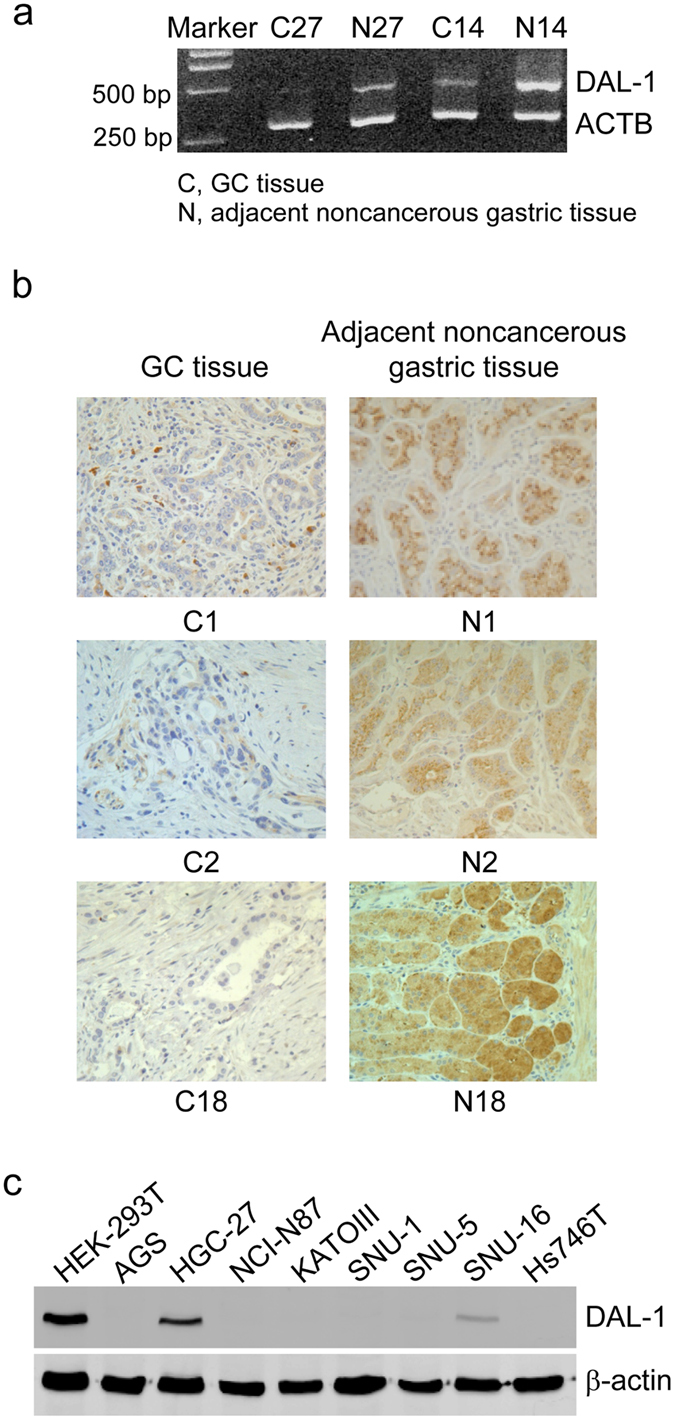
Downregulation of DAL-1 expression in GCs. (**a,b**) DAL-1 expression in GCs and adjacent noncancerous gastric tissues in RT-PCR assay (**a**) (#27 and #14) and in IHC assay (**b**) (#1, #2, #18). Magnification 400×. (**c**) Western blot shows the expression of DAL-1 in GC cells and HEK-293T cells.

To evaluate the expression of DAL-1 in gastric carcinogenesis, we initially analyzed the expression of DAL-1 mRNA and protein in surgically resected GC samples and adjacent noncancerous tissues from the same patients using RT-PCR and IHC methods. RT-PCR results demonstrated that DAL-1 mRNA expression was reduced in 13 of 19 (68.4%) primary GC samples (Fig. 1a, Supplementary Table 1). IHC results revealed that the expression of DAL-1 protein was significantly reduced in GC group compared to the adjacent noncancerous group (20 of 22, 90.9%) (Fig. 1b, Supplementary Table 1 & 2). Additional, we analyzed the DAL-1 expression in GCs and the matched normal samples included in The Cancer Genome Atlas (TCGA) datasets. With the analyses of 333 GC samples and 37 normal gastric samples in TCGA, we found the expression level of DAL-1 in GCs was significantly reduced compared to the matching controls (Supplementary Fig. 1a). The data showed the consistence with our clinical study, that the expression of DAL-1 decreased in GCs. Furthermore, we analyzed the staging data of our clinical samples with the expression levels of DAL-1. We found DAL-1 reduced in various grades and stages, especially in G1, G2, and early stage (stage I) (Supplementary Fig. 1b,c). Meanwhile, through further analysis using the RNA-seq data of DAL-1 in TCGA dataset, we found DAL-1 was obviously reduced in G2 and stage I GCs (Supplementary Fig. 1d,e), which was shown consistent with our clinical samples. It suggested that DAL-1 lost its expression at the beginning of GC development. Moreover, we examined the expression level of DAL-1 protein in eight GC cell lines by Western blot. As shown in Fig. 1c, DAL-1 protein expression was significantly repressed in seven GC cell lines (AGS, NCI-N87, KATOIII, SNU-1, SNU-5, SNU-16, and Hs746T), except HGC-27, compared to that in HEK-293T cells. These data indicate that a reduction in DAL-1 expression may be involved in the development of GC.

### Methylation of the DAL-1 promoter in GCs

**Figure 2:**
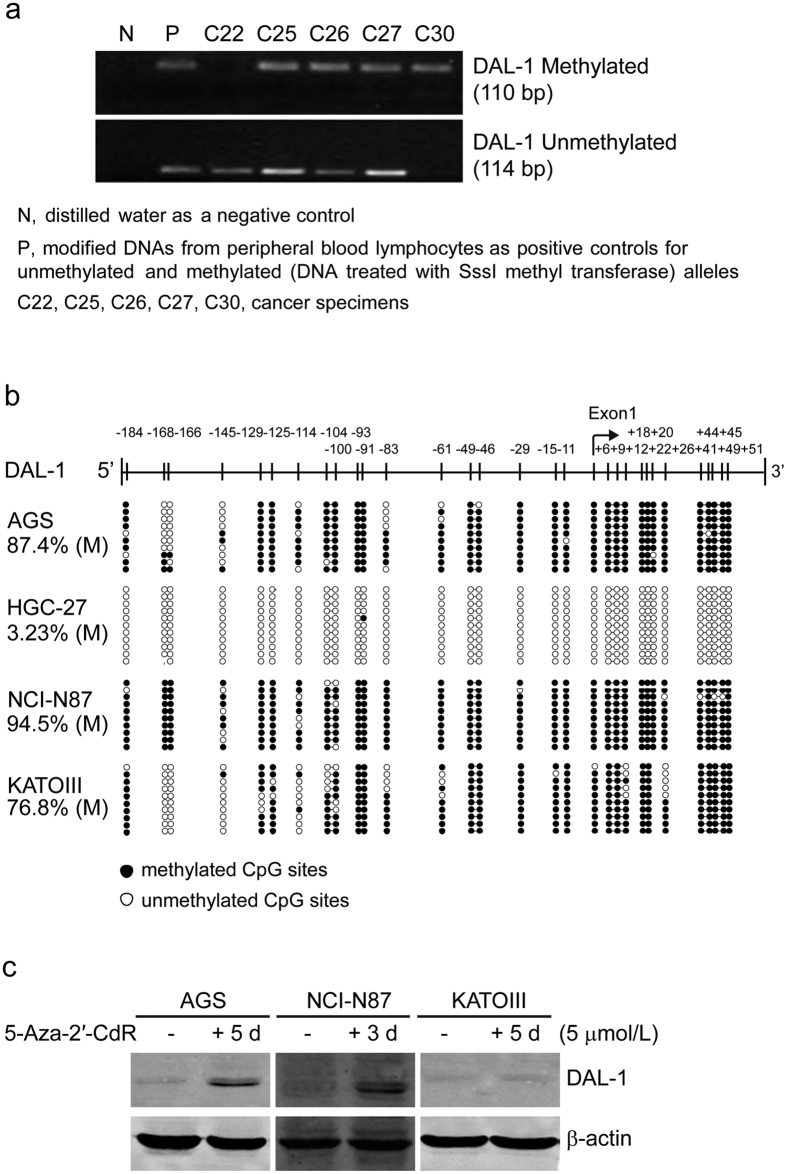
The methylation status of the DAL-1 promoter in GCs. (**a**) The methylation of DAL-1 promoter in GC tissues using the MSP method. Representative cancer specimens showed both methylated and unmethylated alleles for DAL-1. (**b**) The methylation of the DAL-1 promoter in four GC cell lines by BGS. Each row in the grid represents an individual allele of the DAL-1 promoter in one colony sequenced. (**c**) DAL-1 expression in GC cells after demethylation treatment with 5-Aza-2′-CdR.

To investigate whether the downregulation of DAL-1 expression in GCs results from methylation in the promoter region of the gene, we examined the methylation status of the DAL-1 promoter in primary GC tissue samples, and GC cell lines. Thirty-one CpG sites around the transcriptional start site (from −187 bp to +100 bp) were analyzed using the MSP method in 37 paired specimens of primary GC and cancer-adjacent gastric tissues (partially shown in Fig. 2a), including all 19 RT-PCR samples and 21/22 IHC samples aforementioned. We found DAL-1 methylation in 94.6% (35 of 37) of GC cases; in comparison, DAL-1 methylation occurred in 70.3% (26 of 37) cancer-adjacent gastric tissues (Supplementary Table 3). When methylation frequency and DAL-1 expression in GC tissues was compared, we found that the expression of DAL-1 mRNA or protein decreased in 23 of the 35 GC samples in which DAL-1 methylation was apparent (Supplementary Table 1). These results suggest that methylation in the DAL-1 promoter is associated with transcriptional repression of DAL-1 in a subset of GC tissues. However, DAL-1 methylation was also found in six GC samples with normal DAL-1 mRNA expression levels and in two GC samples with normal DAL-1 protein expression levels. The latter phenomenon might be due to a mechanism other than methylation. We then evaluated the methylation frequency of CpG sites located in the DAL-1 promoter region using the BGS method in the GC cell lines AGS, HGC-27, NCI-N87, and KATOIII. We found that these CpG sites were methylated to different degrees (Fig. 2b). The CpG sites in three cell lines were highly methylated; the methylation frequencies were 87.4%, 94.5%, and 76.8% in AGS, NCI-N87, and KATOIII, respectively (Fig. 2b). A decrease in DAL-1 expression was observed in all three cell lines. In contrast, in DAL-1 relatively highly expressed cell line HGC-27, the methylation frequency of the CpG sites in this line was only 3.23% (Fig. 2b). These results indicate that the hypermethylation of DAL-1 CpG sites is strongly correlated with the downregulation of DAL-1 expression in GC cells. To further evaluate whether DAL-1 methylation is a major factor in decreased DAL-1 expression, three hypermethylated DAL-1 cell lines (AGS, NCI-N87, and KATOIII) were treated with the demethylating agent 5-Aza-2′-CdR. As shown in Fig. 2c, the expression of DAL-1 was restored in these cells after 3 or 5 days of demethylation treatment; a methylation-dependent effect on DAL-1 expression was clearly demonstrated. The cells with higher methylation level (AGS, 87.4%, and NCI-N87, 94.5%), were more sensitive to demethylating agent. The results indicated that promoter methylation played an important role in the downregulation of DAL-1 gene expression in these GC cells.

### Aberrant localization of DAL-1 protein in GC cells

**Figure 3:**
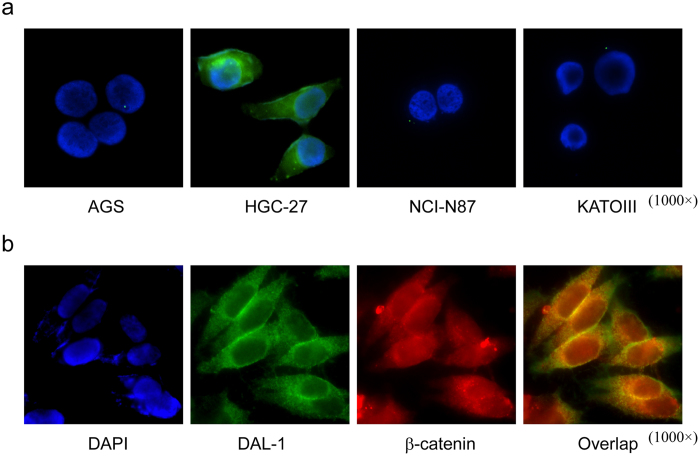
Immunofluorescence localization of DAL-1 in GC cell lines. (**a**) Expression and localization of DAL-1 in AGS, HGC-27, NCI-N87 and KATOIII cells. (**b**) Immunofluorescence co-localization of DAL-1 and β-catenin in HGC-27 cells.

It is known that DAL-1 is expressed at the cell membrane, especially at the interface of cells that are in direct contact with one another in lung and breast cancers^3^,^4^. To detect the cellular localization of the DAL-1 protein in GC cells, we preformed immunofluorescence assays using the following four GC cell lines: AGS, HGC-27, NCI-N87, and KATOIII. As seen in the immunoblotting analysis above, endogenous DAL-1 protein was expressed clearly in HGC-27 GC cells but not in other three GC cell lines (Fig. 3a). Strong signals of DAL-1 protein diffusely distributed throughout the cytoplasm, as well as along the cell membrane (particularly in the cell-cell contact interface of HGC-27 cells). This finding suggests that the pattern of DAL-1 protein expression is aberrant. To validate this finding, we examined whether DAL-1 localizes to the same region as another membrane protein, β-catenin, in HGC-27 cells^15^. Cells were co-incubated with both fluorescein-labeled anti-DAL-1 and anti-β-catenin antibodies; this incubation was followed by DAPI staining (Fig. 3b). DAL-1 was clearly visible at the contact interface between HGC-27 cells and was diffusely dispersed throughout the cytoplasm. β-catenin staining was detected wherever cell-cell contact occurred. DAL-1 and β-catenin signals colocalized at cell-cell contact points, double exposure produced a yellow-orange signal. This complex staining pattern of HGC-27 cells indicates that aberrant DAL-1 protein localization in GC cells may impair its growth-suppressing properties, as DAL-1 may regulate cell-cell attachment or the attachment of cells to another surface.

### Overexpression of DAL-1 impairs the malignancy of GC cells

**Figure 4:**
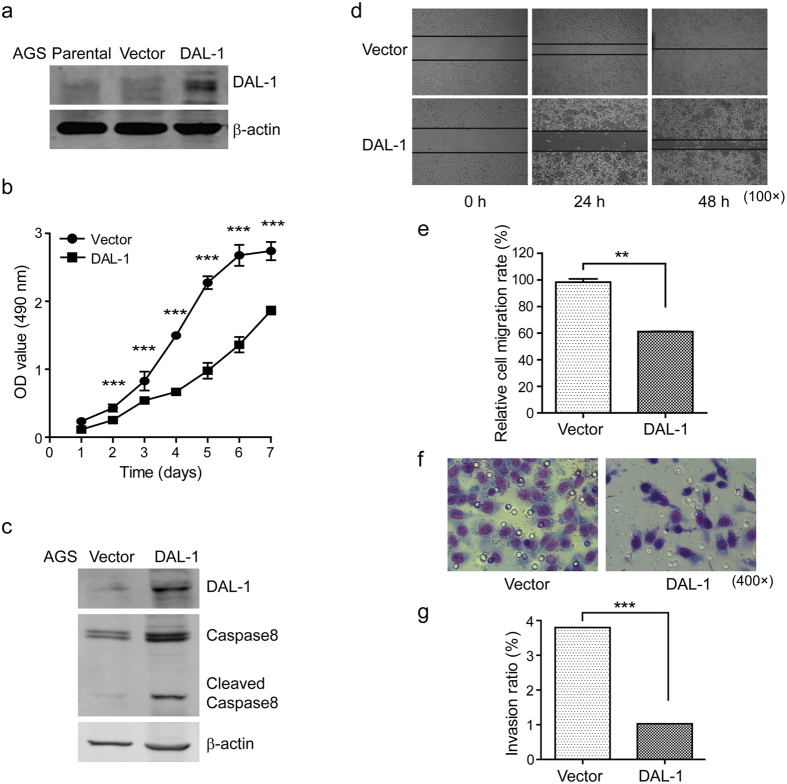
Overexpression of DAL-1 decreases the malignancy potential of AGS cells. (**a**) DAL-1 expression in AGS cells transfected with the DAL-1 and control vector. (**b**) The growth rate of AGS cells. (**c**) The expression of caspase-8 in transfected AGS cells. (**d**) The migrating cells obtained at the indicated time points after wound formation. (**e**) The percentage of the migration rate. (**f**) The invading cells passing through the matrigel-coated membrane. (**g**) The percentage of the invasion rate. **P < 0.01, ***P < 0.001, with *t*-test analysis.

To assess the anticarcinogenic activity of DAL-1 in GC cells, we determined the effect of DAL-1 overexpression on the malignant behavior of AGS cells. AGS cells contain a hypermethylated DAL-1 promoter and lack DAL-1 expression. Stable DAL-1 overexpressing cells and control AGS cells were established by transfection with the pEZ-M68*-*DAL-1 and control vectors (Fig. 4a). Cell proliferation was measured using the MTS assay. Our results demonstrated that the growth rate of DAL-1 overexpressing cells significantly decreased compared to the control cells (Fig. 4b). This finding suggests that DAL-1 suppresses GC cell proliferation. We also checked the expression of caspase-8 in these cells. Through Western blot analysis, we found that overexpression of DAL-1 notably increased caspase-8 expression (Fig. 4c). This result suggests that DAL-1 may be involved in inducing apoptosis in GC cells. Furthermore, we examined whether the upregulation of DAL-1 expression affects AGS cell migration and invasion. By scratching confluent monolayers of DAL-1-transfected and vector control cells, we found that the migration of cancer cells overexpressing DAL-1 decreased compared to control cells (Fig. 4d). This observation was confirmed by the Boyden chamber invasion assay, the overexpression of DAL-1 resulted in a significantly reduced invasion rate compared to control cells (Fig. 4e). In summary, these findings suggest that DAL-1 may repress malignant behaviors in GC cells.

### Downregulation of DAL-1 enhances aggressiveness in GC cells

**Figure 5:**
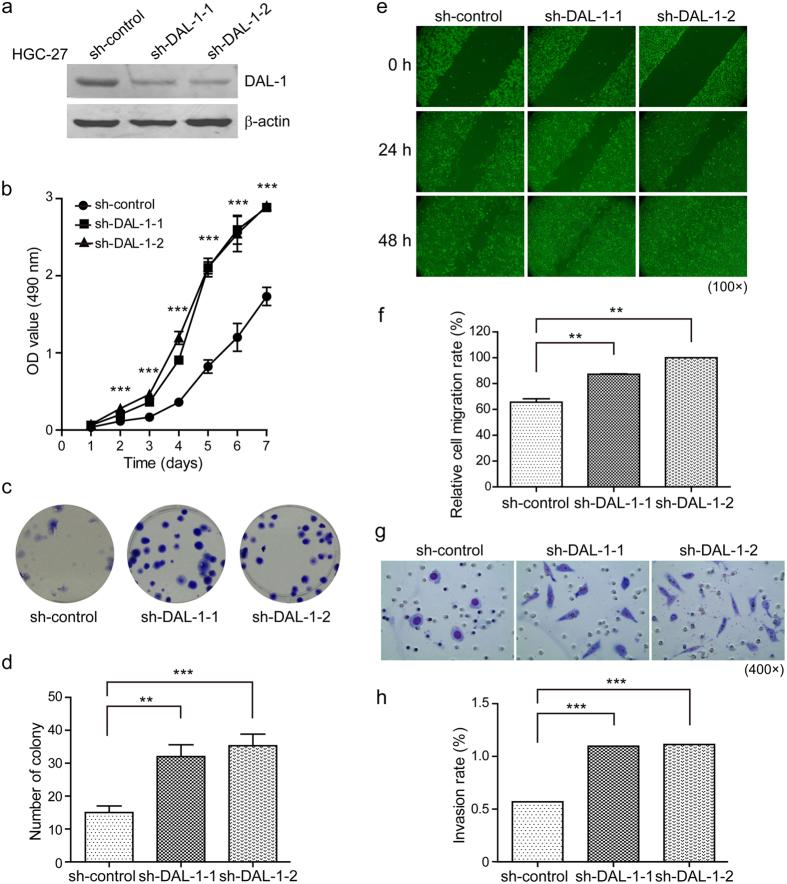
Downregulation of DAL-1 increases the malignancy potential of HGC-27 cells. (**a**) DAL-1 expression in HGC-27 cells transfected with shRNA-DAL-1 and the control vector. (**b**) The growth rate of HGC-27 cells. (**c**) Cell proliferation of HGC-27 cells. (**d**) Quantitive analysis of (**c**). (**e**) The migrating cells obtained at the indicated time points after wound formation. (**f**) The percentage of the migration rate. (**g**) The invading cells passing through the matrigel-coated membrane. (**h**) The percentage of the invasion rate. **P < 0.01, ***P < 0.001, with *ANOVA* (Dunnett’s multiple comparison test).

To further confirm the tumor-suppressing role of DAL-1 in GC cells, we generated a stable knockdown of DAL-1 in HGC-27 cells. HGC-27 cells contain an unmethylated DAL-1 promoter and high DAL-1 expression level. As shown in Fig. 5a, DAL-1 expression decreased in HGC-27 cells stably transfected with shRNA-DAL-1-1 and shRNA-DAL-1-2 compared to control cells. DAL-1 silencing significantly increased cell growth, as measured by MTS, and by clonogenicity in a colony formation assay (Fig. 5b–d). Further, we tested the impact of DAL-1 downregulation on HGC-27 cell migration and invasion using a scratch assay and the Boyden chamber. We observed that the migration of shRNA-DAL-1 cells occurred at a significantly faster rate than that of control cells at 48 h after scratching. When DAL-1 expression was knocked down, more cells invaded through the transmembrane compared to mock-transfected cells (Fig. 5e–h). Together, these results indicate that DAL-1 is an important negative regulator of the biologic phenotype in GC cells.

### DAL-1 regulates the epithelial-mesenchymal process in GC cells

**Figure 6:**
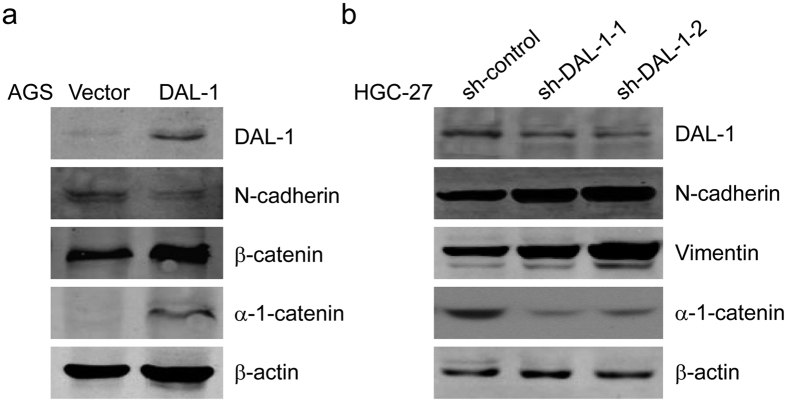
DAL-1 impairs EMT in GC cells. (**a,b**) An immunoblot for the α-1-catenin, β-catenin, N-cadherin and Vimentin in DAL-1-overexpressing AGS (**a**) and DAL-1-knockdown HGC-27 (**b**) cells.

Epithelial to mesenchymal transition (EMT) is a crucial event responsible for cancer cell invasion and metastasis. To determine whether DAL-1 is involved in the EMT process in GC progression, we analyzed the expressions of EMT-related proteins. The four proteins analyzed were α-1-catenin, β-catenin, N-cadherin, and vimentin. Protein expression was detected *via* Western blot in an AGS cell line overexpressing DAL-1 and a HGC-27 cell line in which DAL-1 expression was silenced. Compared to control cells, the expression of the epithelial markers α-1-catenin and β-catenin increased and the expression of the mesenchymal marker N-cadherin decreased in AGS cells with overexpressing DAL-1 (Fig. 6a). Expression of the epithelial marker α-1-catenin decreased and expression of the mesenchymal markers N-cadherin and Vimentin increased in DAL-1-downregulated HGC-27 cells compared to control cells (Fig. 6b). These data suggest that DAL-1 suppresses EMT *via* downregulating the expression of mesenchymal markers and upregulating the expression of epithelial markers in GC cells.

## Discussion

In our previous LOH allelotyping experiment, we identified a deletion region at chromosome band 18p11.3 in 45 sporadic GCs; the DAL-1 gene is localized to this region^1^. This finding encouraged us to further explore the expression pattern of DAL-1 in primary GCs and GC cell lines. We sought to determine the potential link between DAL-1 and GC molecular pathogenesis. The results confirmed that the expression of DAL-1 decreases or was lost in 90.9% (20/22) of primary GCs and 87.5% (7/8) of GCs cell lines. The data of DAL-1 mRNA expression in GC from TCGA was consistent with ours.

The DAL-1 gene harbors a typical DNA sequence that matches the criteria of a CpG island in its upstream region, exon 1, and the beginning of intron 1^6^. It is known that hypermethylation and the loss of expression of DAL-1 are correlated in lung^10^,^16^, breast^11^,^17^, ovarian^18^, prostate^19^, and renal tumors^6^ and meningiomas^9^. In our study, we observed here that DAL-1 was extensively methylated in 75.0% (3/4) of GC cell lines and 94.6% (35/37) of primary GC tissues; this methylation results in a decrease or lack of DAL-1 expression. It is an interesting point that not all the methylation resulted in the decreased expression of DAL-1, 68.4% reduced in RT-PCR assay, and 90.9% reduced in IHC assay. The difference may come from the regulation of transciption and translation, and the limited number of GC cases in this study. In the clinical samples, methylation of the DAL-1 promoter region in the primary GCs was significantly greater than that in the adjacent noncancerous gastric tissues. The results further suggest that methylation contributes to DAL-1 deficiency-induced carcinogenesis. Moreover, the methyltransferase inhibitor 5-Aza-2′-CdR induces significant DAL-1 expression in GC cells where DAL-1 expression is originally repressed. This finding also indicates that methylation is a key factor in DAL-1 gene inactivation. Seemingly, there was no significant change in the expression of DAL-1 in KATOIII cells after 5-Aza-2′-CdR treatment, compared with AGS and NCI-N87 cells. This phenomena might largely due to the different methylation rate among the GC cells. The methylation frequencies were 87.4%, 94.5%, and 76.8% in AGS, NCI-N87, and KATOIII, respectively (Fig. 2b). The cells with higher methylation level, seemed more sensitive to be demethylated by 5-Aza-2′-CdR, which made DAL-1 restored easier: NCI-N87 cells with 94.5% methylation showed expression of DAL-1 restored after 5-Aza-2′-CdR treatment (5 μmol/L) for 3 days; AGS with 87.4% methylation showed DAL-1 restored after the same treatment for 5 days. As for KATOIII cells, its methylation level is 76.8%, which intuitively needs longer time for DAL-1 to restore after 5-Aza-2′-CdR treatment. In addition, the differentiation level of cancer cells could also affect the response to the demethylating agent. KATOIII is a poorly differentiated GC cell line, while AGS and NCI-N87 are well differentiated cell lines^20^,^21^,^22^,^23^. For the above-mentioned reasons, we did not observe obvious restored expression of DAL-1 after 5 days of demethylation treatment in KATOIII cell line.

It has been proposed that promoter methylation of tumor-suppressor genes initially occurs in non-neoplastic gastric epithelia cells. Methylation levels increase with age, gene functions are ultimately silenced, resulting in a defect that may predispose tissues to GC^24^. It is widely accepted that epigenetic alterations are a prerequisite for cancer formation, and these alterations facilitate the accumulation of further genetic abnormalities that result in cancer progression through the clonal expansion of cells with a proliferative advantage^25^. The aberrant methylation of the DAL-1 promoter in histologically normal gastric mucosal cells may be an early epigenetic event in the multistep process of GC progression. We tested and found the evidence for the extensive hypermethylation of the DAL-1 promoter in GC cell lines and primary GCs. Our results suggest that methylation is critical, but sufficient, for DAL-1 downregulation in gastric carcinogenesis. We previously demonstrated that the 18p11.3 LOH is common in primary GCs. We now present findings of promoter hypermethylation and a loss in DAL-1 expression. Together, these works suggest that the DAL-1 gene is inactivated by both promoter methylation and LOH. The coexistence of chromosome instability and hypermethylation indicates that both genetic and epigenetic mechanisms may act in concert to inactivate DAL-1 in primary GCs.

In addition to the phenomenon of DAL-1 downregulation due to promoter hypermethylation, we also observed the mislocalization of expressed DAL-1 in the GC cell line HGC-27. In cancer cells with abnormal DAL-1 subcellular localization, the DAL-1 protein was expressed diffusely within the cytoplasm, except along the cell membrane. Robb *et al.* have shown that the suppression of growth of meningioma cells by 4.1B/DAL-1 requires proper membrane localization. The localization of the U2 domain of Protein 4.1B to the membrane is necessary and sufficient for meningioma growth suppression^26^. Our findings support the idea that this aberrant pattern of subcellular distribution in renal clear cell carcinoma is associated with impaired 4.1B function as a potential tumor suppressor^6^. Additional studies are necessary to prove this concept.

DAL-1 has been previously implicated in cancer cell migration, adhesion, apoptosis, and growth inhibition *in vitro*^3^,^4^,^26^,^27^,^28^,^29^. We therefore examined DAL-1 candidate as a potential suppressor of GC metastasis. DAL-1 is not an adhesion molecule in the classical sense; it acts an anchoring protein, connecting TSLC1 to the actin cytoskeleton and participating in cytoskeleton-associated processes. The loss of DAL-1 expression could, therefore, lead to decreased cell adhesion^14^. It is well known that disrupting the mechanisms that regulate cell adhesion leads to increased growth, invasion, and metastasis. These phenotypic changes were observed in our study when DAL-1 was overexpressed or knocked down in GC cells. Our data demonstrated that the exogenous expression of DAL-1 significantly decreased cell growth, motility, invasiveness, as well as metastasis, the grisly characteristic of malignancy. In contrast, depletion of DAL-1 reverse the phenomena. Thus, epigenetic silencing of DAL-1 contributes to the migratory and invasive phenotype of GC cells. In GC cells overexpressing DAL-1, the expression of the apoptosis-related protein caspase-8 increased. It has been shown previously that the expression of DAL-1 can induce apoptosis in breast cancer MCF-7 cells^4^. Although the mechanisms by which DAL-1 promotes apoptosis remain unclear, our finding supports one study where the overexpression of DAL-1 increased caspase-8 activity in MCF-7 cells^29^.

EMT is an essential morphologic conversion that occurs during embryonic development. EMT has gained more attention in recent years due to its importance in the acquisition of metastatic potential during cancer progression. The perturbation of EMT results in a loss of intracellular adhesion, resulting in cancer progression^30^. We wanted to know whether DAL-1 is involved in the control of EMT in GC. We demonstrated that the overexpression of DAL-1 in AGS cells restrained EMT. The knockdown of DAL-1 in HGC-27 cells promoted EMT; drastic changes in EMT markers were observed, suggesting that DAL-1 may be at least partially responsible for decreased aggressiveness in GC cells.

In summary, the downregulation of DAL-1 is an important event in GC. Downregulation of expression is mainly attributed to methylation of its promoter. In addition, aberrant localization of DAL-1 protein is still not, in our view, a minor matter. Functional analyses of two different GC cells models, either overexpressing DAL-1 or downregulating its expression, demonstrated that DAL-1 acts as a negative modulator of the aggressive cancer phenotype. DAL-1 is therefore a candidate tumor suppressor gene in gastric carcinogenesis. Our studies suggest it is a promising therapeutic target in certain subtypes of GCs.

## Materials and Methods

### Cell lines, cell culture, and 5-Aza-2′-deoxycytidine (5-Aza-2′-CdR) treatment

The human GC cell lines AGS, NCI-N87, KATOIII, SNU-1, SNU-5, SNU-16, and Hs746T and the human embryonic kidney cell line HEK-293T were purchased from the American Type Culture Collection (ATCC, Manassas, VA, USA). The human GC cell line HGC-27 was obtained from the Cell Resources Center of Shanghai Life Sciences, Chinese Academy of Sciences (Shanghai, China). The cells were authenticated by the Beijing Microread Genetics (Beijing, China) using short tandem repeat (STR) analysis. All cells were routinely maintained. AGS, NCI-N87, and KATOIII cells (1 × 10^6^/mL) were grown in either the presence or absence of the demethylating agent 5-Aza-2′-CdR (5 μmol/L, Sigma, St. Louis, MO, USA) for 3 to 5 days, and then checked with the expression of DAL-1.

### Tissue specimens

Thirty-eight pairs of cancerous and adjacent noncancerous tissues from GC patients were collected after surgical resection from the First and the Second Affiliated Hospitals of Harbin Medical University (Harbin, China) in 2005 and 2006. Specimens were snap-frozen immediately after resection. Of the 38 patients, 22 pairs of formalin-fixed, paraffin-embedded specimens (5-μm thickness) were also obtained. Appropriate informed consent was obtained from the patients. The study was approved by the Ethics Committee of Harbin Medical University. The methods were carried out in accordance with the approved guidelines.

### Immunoblotting analysis and immunofluorescence

For immunoblotting, cells were lysed in RIPA buffer, followed by centrifugation, and subjected to SDS-PAGE and transferred to polyvinylidene difluoride (PVDF) membranes. The membranes were immunoblotted with various primary antibodies [DAL-1 (Abnove, Taiwan), α-1-catenin (Abcam, Cambridge, MA, USA), β-catenin (Abcam), N-cadherin (BD Biosciences, Bedford, MA, USA), vimentin (BD Biosciences) and β-actin (Zhongshan Biotech Co., Beijing, China)], followed by the corresponding fluorescent-conjugated anti-mouse or anti-rabbit antibodies (Rockland Immunochemicals Inc., Gilbertsville, PA, USA). The fluorescence signals were visualized using an Odyssey Infrared Imaging System (Li-COR, USA). For immunofluorescence, cells were seeded onto coverslips in six-well plates, and then were fixed in 4% paraformaldehyde. The coverslips were coated with primary antibodies against DAL-1 and β-catenin, followed by incubation with the anti-mouse or anti-rabbit antibodies respectively, and 4′,6-diamidino-2-phenylindole (DAPI) staining. Images were obtained using a Leica DM5000B microscope (Leica Microsystems, Solms, Germany).

### Reverse transcriptase-polymerase chain reaction (RT-PCR)

Total RNA was isolated using the TRIzol Reagent Kit (Invitrogen, Auckland, NZ, USA) according to the manufacturer’s instructions, and then reverse-transcribed to cDNA using the Reverse TranScription System (Promega, Madison, WI, USA), and was amplified by PCR. The cDNA expression of DAL-1 was normalized against ACTB. All measurements were performed in triplicate. The primers were designed using Primer 3.0 software. The primers used for DAL-1 were as follows: 5′-CATTCACAGGCATTAAAGGG-3′ and 5′-CCGTGATGACTATTCGCTTC-3′; for ACTB were 5′-ACTCTTCCAGCCTTCCTTCC-3′ and 5′-CATACTCCTGCTTGCTGATCC-3′. The PCR products were then separated by agarose gel electrophoresis.

### Immunohistochemistry (IHC)

Immunohistochemical staining of DAL-1 (using the anti-DAL-1 antibody, Santa Cruz Biotechnology, Japan) was performed on 22 paired GC tissues and adjacent noncancerous gastric tissues as previously described^31^. Global DAL-1 staining was scored as follows: 0 (no staining), 1 (focused or weak), 2 (moderate, 25–50%), 3 (strong, 25–50%), and 4 (strong, >50%).

### Bisulfite genomic sequencing (BGS)

Genomic DNA from the GC cell lines was isolated using a QIAmp DNA mini Kit (Qiagen, Valencia, CA, USA), and was modified using a Methylamp™ DNA Modification Kit (Epigentek, USA). A search for CpG islands in the promoter region of DAL-1 (GenBank accession NM_012307) revealed a CpG island that was 223 bp long. and located at nt −187 ∼ nt +100 from the transcription start site. Modified DNA from the cell lines was subjected to PCR to amplify a 288 bp DNA fragment (containing 31 CpG sites). The following pair of primers was used: 5′-TTTATGTAATTGTTTTGAAGTATTG -3′ and 5′-TTACCTAAAATCAACAAAAAACCC-3′. The purified PCR products were inserted into the pEASY-T3 vector, and then transformed. At least 10 bacterial colonies were picked for each GC cell line, followed by sequencing confirmation (Invitrogen Biotechnology Co. Ltd, Shanghai, China). The sequences were analyzed for CpG site methylation status using BIQ Analyzer software (Max-Planck-Institut Informatik, Munich, Bavaria, Germany).

### Methylation-specific PCR (MSP)

Bisulfite-treated DNA was amplified using primers designed to anneal specifically to methylated or unmethylated bisulfite-modified DNA sequences within genes, as reported previously^32^. These primer sequences were as follows: 5′-AGGTTGGTTTTTTTTGTATGGTT-3′ and 5′-AACCCAAAACTACTCACCACT-3′ for detecting unmethylated sequences, or 5′-TTGGTTTTTTTCGTACGGTT-3′ and 5′-AACCCAAAACTACTCGCCG-3′ for detecting methylated sequences. The MSP products were then separated by agarose gel electrophoresis.

### Generation of stable GC cells overexpressing DAL-1 or expressing shRNA targeting DAL-1

AGS cells were transfected with the pEZ-M68-DAL-1 vector or a control vector (FulenGen, Guangzhou, China) using the Lipofectamine 2000 transfection reagent (Invitrogen). Cells were incubated in the presence of 1 μg/mL puromycin. The expression of DAL-1 protein was confirmed by Western blot. The short hairpin RNA (shRNA) oligonucleotides targeting DAL-1 were synthesized by Genechem (Shanghai, China). The sequences of DAL-1 shRNAs were as follows: 5′-AAGCGTTTATGAAAGTAT-3′ (shRNA-1) and 5′-ATCACATTTCAGAAACTT-3′ (shRNA-2). The recombinant GV102-shRNA-DAL-1 plasmids and the control vector (control) were transfected into HGC-27 cells. Stable cell lines were established after G418 (600 μg/mL) selection. The knockdown of DAL-1 expression was confirmed by Western blot.

### Cell viability, colony formation assays, migration and invasion assay

Cells viability was measured using MTS solution (Promega) over the course of 7 days. Colony formation assay was performed by growing the cells for 14 days, and staining the cell colonies with Giemsa to measure the ability of cells to proliferate. Cells migration were examined at 0, 24 h, and 48 h post scratch. The relative migration rate was determined by measuring the average area of the wound gap. Cell invasion ability was assessed with Boyden chamber (BD Biosciences) according to the manufacturer’s instructions. The results were expressed as the percentage of cells that had migrated relative to the total number of seeded cells.

## Additional Information

**How to cite this article**: Wang, H. *et al.* Aberrant expression of the candidate tumor suppressor gene DAL-1 due to hypermethylation in gastric cancer. *Sci. Rep.* **6**, 21755; doi: 10.1038/srep21755 (2016).

## Supplementary Material

Supplementary Information
