# Supplementary material for: Aberrant expression of the candidate tumor suppressor gene DAL-1 due to hypermethylation in gastric cancer
Source: Sci Rep. 2016 Feb 29;6:21755. doi: 10.1038/srep21755 (PMC4770418; doi:10.1038/srep21755)
Supplement: Supplementary Information [file srep21755-s1.pdf]

Aberrant expression of the candidate tumor suppressor gene DAL-1 due to hypermethylation in gastric cancer

Running title: DAL-1 deficiency due to hypermethylation in GC

Hao Wang[3,#], Man Xu[1,#], Xiaobo Cui[2], Yixin Liu[4], Yi Zhang[2], Yu Sui[2], Dong Wang[1], Lei Peng[1], Dexu Wang[1], Jingcui Yu[1*]

[1]Scientific Research Centre, the Second Affiliated Hospital of Harbin Medical University, Harbin 150081, China; [2]Laboratory of Medical Genetics, Harbin Medical University, Harbin 150081, China; [3]Department of Hepatopancreatobiliary Surgery, the Second Affiliated Hospital of Harbin Medical University, Harbin 150081, China; [4]Computer teaching and research section, School of Basic Medical Science, Harbin Medical University.

[#]These authors contributed equally to this work.

[*]Corresponding author: Professor Jingcui Yu, Scientific Research Centre, the Second Affiliated Hospital of Harbin Medical University, 246 Xuefu Road, Nangang District, Harbin 150081, China. Phone: +86-451-86605908; email: yujingcui@ems.hrbmu.edu.cn.

Supplementary Table 1. Relative expresssion level and methylation status of DAL-1 in GC and adjacent noncancerous gastric tissues

Tissue No.	Gender	Age	Grade	Stage	Relative expression level of DAL-1 in RT-PCR assay (intensity scale of gel band)		Relative expression level of DAL-1 in IHC assay		DAL-1 methylation status			
									Cancerous		Noncancerous	
					Cancerous	Noncancerous	Cancerous	Noncancerous	Methylated	Unmethylated	Methylated	Unmethylated
1	female	49	G3	II	0.61	0.18	1	4	+	+	+	+
2	male	50	G3	II	N/A	N/A	1	4	+	+	+	+
3	male	54	G3	III	0.98	0.73	0	4	+	+	-	+
4	female	64	G3	III	0.92	0.99	0	0	+	+	-	+
5	female	64	G2	Ⅱ	0.34	0.76	0	4	+	+	+	+
6	male	65	G3	Ⅱ	1.04	1.20	1	4	-	-	-	+
7	male	54	G3	III	N/A	N/A	2	3	+	+	-	+
8	male	54	G3	III	1.07	0.57	1	1	+	+	-	+
9	male	60	G3	III	N/A	N/A	0	4	+	+	-	+
10	female	69	G3	Ⅱ	N/A	N/A	N/A	N/A	+	+	+	+
11	female	69	G3	III	N/A	N/A	N/A	N/A	+	+	+	+
12	male	61	G2	Ⅰ	0.37	1.04	N/A	N/A	+	+	+	+
13	female	36	G3	III	0.31	0.00	0	3	+	-	+	+
14	male	58	G2	Ⅰ	0.64	1.06	1	3	+	-	+	-
15	female	52	G3	Ⅱ	N/A	N/A	N/A	N/A	+	+	+	+
16	male	49	G3	Ⅰ	N/A	N/A	2	4	+	+	+	+
17	male	76	G2	Ⅱ	N/A	N/A	1	3	+	+	+	+
18	male	34	G3	Ⅲ	0.01	0.07	0	4	+	+	+	+
19	male	69	G1	II	N/A	N/A	0	4	+	+	+	+
20	male	63	G1	II	0	0.23	0	3	+	+	+	+

Tissue No.	Gender	Age	Grade	Stage	Relative expression level of DAL-1 in RT-PCR assay (intensity scale of gel band)		Relative expression level of DAL-1 in IHC assay		DAL-1 methylation status			
									Cancerous		Noncancerous	
					Cancerous	Noncancerous	Cancerous	Noncancerous	Methylated	Unmethylated	Methylated	Unmethylated
21	female	52	G3	III	2.05	0.76	0	3	+	+	+	+
22	male	71	G3	II	N/A	N/A	0	2	-	+	-	+
23	male	55	G3	II	N/A	N/A	N/A	N/A	+	+	+	+
24	female	65	G2	II	0.85	1.13	N/A	N/A	+	+	+	+
25	male	70	G3	III	0.97	0.56	0	3	+	+	+	-
26	male	71	G3	II	0.43	0.99	0	3	+	+	+	-
27	female	56	G3	I	0.36	0.69	0	4	+	+	-	+
28	male	81	G3	III	N/A	N/A	N/A	N/A	+	+	-	+
29	male	76	G3	III	N/A	N/A	N/A	N/A	+	+	-	+
30	female	48	G1	I	N/A	N/A	N/A	N/A	+	-	+	+
31	male	60	G2	II	0.57	0.72	N/A	N/A	+	+	+	+
32	male	68	G3	III	N/A	N/A	N/A	N/A	+	+	+	+
33	female	37	G2	III	N/A	N/A	N/A	N/A	+	-	+	+
34	male	58	G2	I	N/A	N/A	N/A	N/A	+	+	+	+
35	male	63	G3	III	0.67	0.94	N/A	N/A	+	+	+	+
36	male	64	G3	III	N/A	N/A	N/A	N/A	+	+	-	+
37	female	62	G3	III	0.88	1.49	N/A	N/A	+	+	+	+
38	female	35	G3	III	N/A	N/A	0	4	N/A	N/A	N/A	N/A

Supplementary Table 2 Statistical analysis for IHC analysis of DAL-1 expression in tissues

Rank	GC tissues	Adjacent noncancerous gastric tissue	*P-value*
0	14	1	
1	6	1	
2	2	1	<0.0001***
3	0	8	
4	0	11	

****P*<0.001, according to Kruskal-Wallis Test

Supplementary Table 3 Statistical analysis for DAL-1 methylation status in tissues

Group	DAL-1 methylation status		*P-value*
	Methylated (n=37)	Unmethylated (n=37)	
GC tissues	35	2	0.0060**
Adjacent noncancerous gastric tissue	26	11	

***P*<0.01, according to Fisher's exact test

Supplementary Figure 1

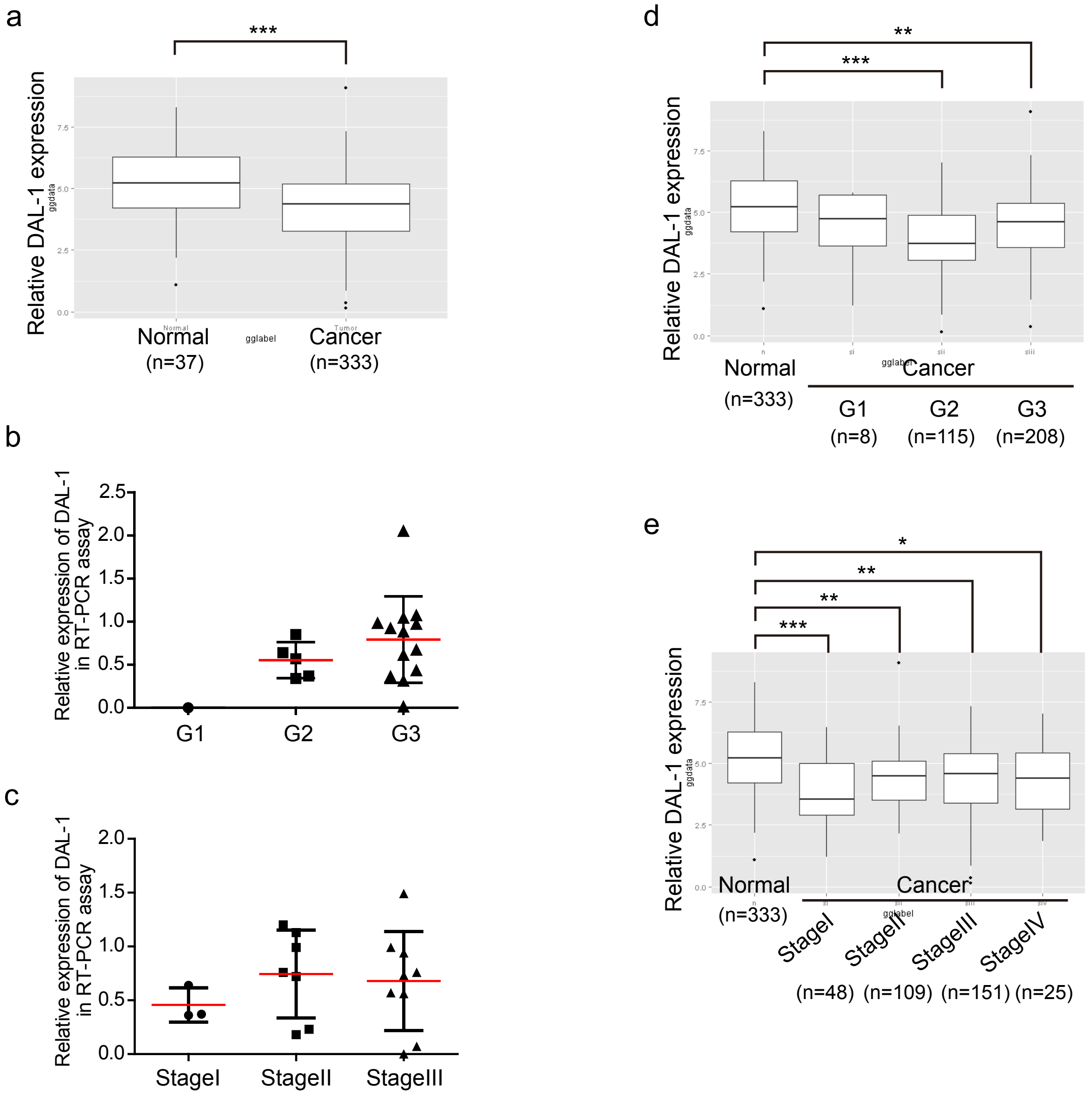

Supplementary Figure Legends

Figure 1. Decreased expression of DAL-1 in gastric cancers (GCs). (a) The expression levels of DAL-1 in GCs and the matched normal gastric samples in The Cancer Genome Atlas (TCGA) datasets. ***P<0.001, with *t*-test analysis. (b) and (c) The expression levels of DAL-1 in different grade or stage GCs of our own primary samples. The data are described as mean ± standard derivation (SD). (d) and (e) The expression levels of DAL-1 in normal gastric tissues and the different grade or stage GCs in TCGA datasets. **P<0.01, ***P<0.001, by *AN*OVA and Dunnett's multiple comparison post-test.
